# 1-(2,3,4-Trihydroxy­benzyl­idene)-4-ethyl­thio­semicarbazide

**DOI:** 10.1107/S1600536810014078

**Published:** 2010-04-24

**Authors:** Hana Bashir Shawish, M. Jamil Maah, Seik Weng Ng

**Affiliations:** a Department of Chemistry, University of Malaya, 50603 Kuala Lumpur, Malaysia

## Abstract

In the title mol­ecule, C_10_H_13_N_3_O_3_S, the thio­semicarbazide =N—NH—C(=S)—NH– fragment is twisted with respect to the aromatic ring [dihedral angle = 20.5 (1)°]. A weak N—H⋯S hydrogen bond [3.480 (1) Å] links two mol­ecules about a center of inversion to generate a ring. The hydr­oxy groups are engaged in inter­molecular hydrogen bonding; the O—H⋯O and O—H⋯S hydrogen bonds generate a layer motif.

## Related literature

For the crystal structures of 3,4-dihydroxy­benzaldehyde 4-ethyl­thio­semicarbazone and 2,4-dihydroxy­benzaldehyde 4-ethyl­thio­semicarbazone, see: Kayed *et al.* (2008 ▶); Tan *et al.* (2008 ▶).
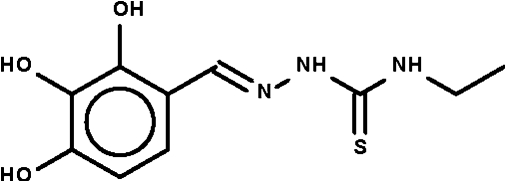


## Experimental

### 

#### Crystal data

C_10_H_13_N_3_O_3_S
*M* *_r_* = 255.29
Monoclinic, 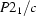

*a* = 7.5668 (5) Å
*b* = 14.6754 (10) Å
*c* = 10.8700 (7) Å
β = 104.711 (1)°
*V* = 1167.50 (13) Å^3^
*Z* = 4
Mo *K*α radiation
μ = 0.28 mm^−1^
*T* = 100 K
0.30 × 0.20 × 0.10 mm

#### Data collection

Bruker SMART APEX diffractometer
Absorption correction: multi-scan (*SADABS*; Sheldrick, 1996 ▶) *T*_min_ = 0.921, *T*_max_ = 0.973
10870 measured reflections
2660 independent reflections
2364 reflections with *I* > 2σ(*I*)
*R*_int_ = 0.026

#### Refinement

*R*[*F* ^2^ > 2σ(*F* ^2^)] = 0.029
*wR*(*F* ^2^) = 0.082
*S* = 1.04
2660 reflections
174 parameters
5 restraints
H atoms treated by a mixture of independent and constrained refinement
Δρ_max_ = 0.34 e Å^−3^
Δρ_min_ = −0.22 e Å^−3^

### 

Data collection: *APEX2* software (Bruker, 2009 ▶); cell refinement: *SAINT* (Bruker, 2009 ▶); data reduction: *SAINT*; program(s) used to solve structure: *SHELXS97* (Sheldrick, 2008 ▶); program(s) used to refine structure: *SHELXL97* (Sheldrick, 2008 ▶); molecular graphics: *X-SEED* (Barbour, 2001 ▶); software used to prepare material for publication: *publCIF* (Westrip, 2010 ▶).

## Supplementary Material

Crystal structure: contains datablocks global, I. DOI: 10.1107/S1600536810014078/pk2241sup1.cif

Structure factors: contains datablocks I. DOI: 10.1107/S1600536810014078/pk2241Isup2.hkl

Additional supplementary materials:  crystallographic information; 3D view; checkCIF report

## Figures and Tables

**Table 1:** Hydrogen-bond geometry (Å, °)

*D*—H⋯*A*	*D*—H	H⋯*A*	*D*⋯*A*	*D*—H⋯*A*
O1—H1*O*⋯O2	0.83 (1)	2.26 (2)	2.717 (1)	115 (2)
O1—H1*O*⋯S1^i^	0.83 (1)	2.55 (1)	3.291 (1)	150 (2)
O2—H2*O*⋯O3	0.84 (1)	2.31 (2)	2.745 (1)	112 (2)
O2—H2*O*⋯O1^ii^	0.84 (1)	2.07 (1)	2.832 (1)	151 (2)
O3—H3*O*⋯S1^iii^	0.84 (1)	2.36 (1)	3.189 (1)	170 (2)
N2—H2*N*⋯S1^iv^	0.87	2.62	3.480 (1)	171

Symmetry codes: (i) 
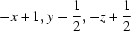
; (ii) 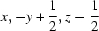; (iii) 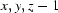; (iv) 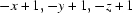.

**Fig. 1.**
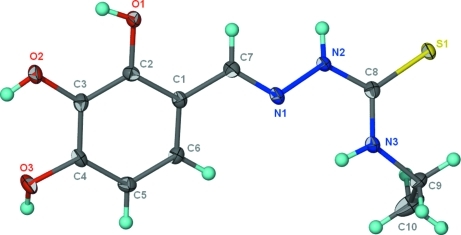
Thermal ellipsoid plot (Barbour, 2001) of C10H13N3O3S at the 70% probability level; hydrogen atoms are drawn as spheres of arbitrary radius.

## supplementary crystallographic information

### Experimental

2,3,4-Trihydroxybenzaldehyde (1.54 g, 10 mmol) and 4-ethylthiosemicarbazide (1.19 g, 1 mmol) were heated in ethanol (20 ml) for 2 hours; acetic acid (0.5 ml) was also added. A brown solid separated from the cool solution; this was recrystallized from methanol.

### Refinement

Carbon-bound H-atoms were placed in calculated positions (C—H 0.95 to 0.98 Å) and were included in the refinement in the riding model approximation, with *U*(H) set to 1.2 or 1.5*U*(C_Me_).

The amino and hydroxy H-atoms were located in a difference Fourier map, and were refined with distance restraints of N–H 0.86±0.01 and O–H 0.84±0.01 Å; their temperature factors were freely refined.

### Crystal data

**Table:**

C_10_H_13_N_3_O_3_S	*F*(000) = 536
*M**_r_* = 255.29	*D*_x_ = 1.452 Mg m^−^^3^
Monoclinic, *P*2_1_/*c*	Mo *K*α radiation, λ = 0.71073 Å
Hall symbol: -P 2ybc	Cell parameters from 5730 reflections
*a* = 7.5668 (5) Å	θ = 2.4–28.3°
*b* = 14.6754 (10) Å	µ = 0.28 mm^−^^1^
*c* = 10.8700 (7) Å	*T* = 100 K
β = 104.711 (1)°	Prism, colorless
*V* = 1167.50 (13) Å^3^	0.30 × 0.20 × 0.10 mm
*Z* = 4	

### Data collection

**Table:**

Bruker SMART APEX diffractometer	2660 independent reflections
Radiation source: fine-focus sealed tube	2364 reflections with *I* > 2σ(*I*)
graphite	*R*_int_ = 0.026
ω scans	θ_max_ = 27.5°, θ_min_ = 2.4°
Absorption correction: multi-scan (*SADABS*; Sheldrick, 1996)	*h* = −9→9
*T*_min_ = 0.921, *T*_max_ = 0.973	*k* = −19→19
10870 measured reflections	*l* = −14→13

### Refinement

**Table:**

Refinement on *F*^2^	Primary atom site location: structure-invariant direct methods
Least-squares matrix: full	Secondary atom site location: difference Fourier map
*R*[*F*^2^ > 2σ(*F*^2^)] = 0.029	Hydrogen site location: inferred from neighbouring sites
*wR*(*F*^2^) = 0.082	H atoms treated by a mixture of independent and constrained refinement
*S* = 1.04	*w* = 1/[σ^2^(*F*_o_^2^) + (0.0437*P*)^2^ + 0.4981*P*] where *P* = (*F*_o_^2^ + 2*F*_c_^2^)/3
2660 reflections	(Δ/σ)_max_ = 0.001
174 parameters	Δρ_max_ = 0.34 e Å^−^^3^
5 restraints	Δρ_min_ = −0.21 e Å^−^^3^

### Fractional atomic coordinates and isotropic or equivalent isotropic displacement parameters (Å^2^)

**Table:**

	*x*	*y*	*z*	*U*_iso_*/*U*_eq_	
S1	0.70929 (4)	0.59538 (2)	0.58991 (3)	0.01292 (10)	
O1	0.50792 (13)	0.28115 (6)	0.03429 (8)	0.0153 (2)	
O2	0.54351 (14)	0.26317 (6)	−0.20701 (9)	0.0176 (2)	
O3	0.73079 (14)	0.39064 (6)	−0.30899 (9)	0.0167 (2)	
N1	0.73526 (15)	0.49383 (7)	0.26113 (10)	0.0133 (2)	
N2	0.69204 (15)	0.50533 (7)	0.37631 (10)	0.0132 (2)	
N3	0.91494 (16)	0.61386 (8)	0.42338 (11)	0.0150 (2)	
C1	0.66760 (17)	0.42305 (8)	0.05845 (12)	0.0114 (2)	
C2	0.59670 (17)	0.34728 (8)	−0.01603 (12)	0.0114 (2)	
C3	0.61719 (17)	0.33861 (8)	−0.13940 (12)	0.0122 (2)	
C4	0.71179 (18)	0.40505 (8)	−0.18813 (12)	0.0123 (2)	
C5	0.78291 (17)	0.48082 (9)	−0.11516 (12)	0.0134 (3)	
H5	0.8474	0.5261	−0.1486	0.016*	
C6	0.75918 (17)	0.48975 (8)	0.00612 (12)	0.0127 (2)	
H6	0.8059	0.5421	0.0550	0.015*	
C7	0.64158 (17)	0.43394 (8)	0.18633 (12)	0.0124 (2)	
H7	0.5559	0.3969	0.2138	0.015*	
C8	0.77826 (17)	0.57177 (8)	0.45530 (12)	0.0118 (2)	
C9	1.01416 (19)	0.69298 (9)	0.48851 (13)	0.0168 (3)	
H9A	1.1456	0.6869	0.4917	0.020*	
H9B	1.0020	0.6951	0.5770	0.020*	
C10	0.9416 (2)	0.78045 (10)	0.42107 (17)	0.0283 (4)	
H10A	1.0080	0.8323	0.4678	0.042*	
H10B	0.8112	0.7863	0.4171	0.042*	
H10C	0.9584	0.7795	0.3346	0.042*	
H1O	0.472 (3)	0.2409 (11)	−0.0195 (16)	0.039 (6)*	
H2O	0.554 (3)	0.2673 (15)	−0.2817 (11)	0.045 (6)*	
H3O	0.731 (3)	0.4420 (9)	−0.343 (2)	0.047 (6)*	
H2N	0.5992 (17)	0.4785 (11)	0.3933 (15)	0.021 (4)*	
H3N	0.936 (2)	0.5975 (11)	0.3531 (11)	0.023 (4)*	

### Atomic displacement parameters (Å^2^)

**Table:**

	*U*^11^	*U*^22^	*U*^33^	*U*^12^	*U*^13^	*U*^23^
S1	0.01775 (18)	0.01251 (16)	0.00958 (16)	−0.00113 (11)	0.00547 (12)	−0.00111 (10)
O1	0.0228 (5)	0.0130 (4)	0.0113 (4)	−0.0065 (4)	0.0064 (4)	−0.0022 (3)
O2	0.0281 (5)	0.0147 (5)	0.0114 (5)	−0.0064 (4)	0.0078 (4)	−0.0041 (4)
O3	0.0259 (5)	0.0153 (5)	0.0110 (5)	0.0000 (4)	0.0089 (4)	0.0007 (3)
N1	0.0157 (5)	0.0150 (5)	0.0104 (5)	−0.0001 (4)	0.0056 (4)	−0.0018 (4)
N2	0.0155 (5)	0.0149 (5)	0.0109 (5)	−0.0040 (4)	0.0063 (4)	−0.0028 (4)
N3	0.0185 (6)	0.0158 (5)	0.0126 (5)	−0.0046 (4)	0.0075 (4)	−0.0047 (4)
C1	0.0116 (6)	0.0122 (5)	0.0103 (6)	0.0016 (4)	0.0024 (4)	0.0003 (4)
C2	0.0118 (6)	0.0105 (5)	0.0124 (6)	0.0007 (4)	0.0038 (5)	0.0015 (4)
C3	0.0144 (6)	0.0109 (6)	0.0109 (6)	0.0011 (5)	0.0023 (5)	−0.0012 (4)
C4	0.0137 (6)	0.0144 (6)	0.0096 (6)	0.0034 (5)	0.0042 (5)	0.0009 (4)
C5	0.0132 (6)	0.0129 (6)	0.0144 (6)	−0.0004 (5)	0.0040 (5)	0.0027 (5)
C6	0.0127 (6)	0.0118 (6)	0.0126 (6)	−0.0003 (5)	0.0016 (5)	−0.0010 (5)
C7	0.0138 (6)	0.0111 (6)	0.0128 (6)	0.0005 (5)	0.0042 (5)	0.0002 (4)
C8	0.0140 (6)	0.0104 (5)	0.0110 (6)	0.0015 (5)	0.0028 (5)	0.0011 (4)
C9	0.0182 (7)	0.0164 (6)	0.0167 (6)	−0.0067 (5)	0.0060 (5)	−0.0043 (5)
C10	0.0201 (8)	0.0167 (7)	0.0444 (10)	−0.0032 (6)	0.0012 (7)	0.0005 (6)

### Geometric parameters (Å, °)

**Table:**

S1—C8	1.7092 (13)	C1—C6	1.4007 (17)
O1—C2	1.3707 (15)	C1—C7	1.4613 (17)
O1—H1O	0.827 (9)	C2—C3	1.3944 (17)
O2—C3	1.3669 (15)	C3—C4	1.3914 (17)
O2—H2O	0.838 (9)	C4—C5	1.3923 (17)
O3—C4	1.3738 (15)	C5—C6	1.3811 (17)
O3—H3O	0.841 (9)	C5—H5	0.9500
N1—C7	1.2819 (16)	C6—H6	0.9500
N1—N2	1.3824 (14)	C7—H7	0.9500
N2—C8	1.3523 (16)	C9—C10	1.511 (2)
N2—H2N	0.866 (9)	C9—H9A	0.9900
N3—C8	1.3244 (17)	C9—H9B	0.9900
N3—C9	1.4639 (16)	C10—H10A	0.9800
N3—H3N	0.853 (9)	C10—H10B	0.9800
C1—C2	1.3996 (17)	C10—H10C	0.9800
			
C2—O1—H1O	109.3 (15)	C6—C5—H5	120.2
C3—O2—H2O	109.6 (15)	C4—C5—H5	120.2
C4—O3—H3O	107.5 (15)	C5—C6—C1	121.26 (11)
C7—N1—N2	115.99 (11)	C5—C6—H6	119.4
C8—N2—N1	118.43 (11)	C1—C6—H6	119.4
C8—N2—H2N	118.7 (11)	N1—C7—C1	119.47 (11)
N1—N2—H2N	122.0 (11)	N1—C7—H7	120.3
C8—N3—C9	125.59 (11)	C1—C7—H7	120.3
C8—N3—H3N	116.0 (12)	N3—C8—N2	116.93 (11)
C9—N3—H3N	117.8 (12)	N3—C8—S1	123.94 (10)
C2—C1—C6	118.47 (11)	N2—C8—S1	119.13 (10)
C2—C1—C7	120.84 (11)	N3—C9—C10	111.14 (11)
C6—C1—C7	120.66 (11)	N3—C9—H9A	109.4
O1—C2—C3	120.30 (11)	C10—C9—H9A	109.4
O1—C2—C1	119.11 (11)	N3—C9—H9B	109.4
C3—C2—C1	120.59 (11)	C10—C9—H9B	109.4
O2—C3—C4	122.81 (11)	H9A—C9—H9B	108.0
O2—C3—C2	117.46 (11)	C9—C10—H10A	109.5
C4—C3—C2	119.72 (11)	C9—C10—H10B	109.5
O3—C4—C3	116.43 (11)	H10A—C10—H10B	109.5
O3—C4—C5	123.27 (11)	C9—C10—H10C	109.5
C3—C4—C5	120.30 (12)	H10A—C10—H10C	109.5
C6—C5—C4	119.63 (12)	H10B—C10—H10C	109.5
			
C7—N1—N2—C8	−175.71 (11)	O3—C4—C5—C6	−179.39 (12)
C6—C1—C2—O1	179.53 (11)	C3—C4—C5—C6	0.03 (19)
C7—C1—C2—O1	−2.47 (18)	C4—C5—C6—C1	1.21 (19)
C6—C1—C2—C3	0.05 (18)	C2—C1—C6—C5	−1.25 (19)
C7—C1—C2—C3	178.06 (11)	C7—C1—C6—C5	−179.26 (12)
O1—C2—C3—O2	0.95 (18)	N2—N1—C7—C1	174.75 (11)
C1—C2—C3—O2	−179.58 (11)	C2—C1—C7—N1	167.00 (12)
O1—C2—C3—C4	−178.31 (11)	C6—C1—C7—N1	−15.04 (18)
C1—C2—C3—C4	1.16 (19)	C9—N3—C8—N2	173.77 (12)
O2—C3—C4—O3	−0.96 (18)	C9—N3—C8—S1	−7.36 (19)
C2—C3—C4—O3	178.26 (11)	N1—N2—C8—N3	−8.18 (17)
O2—C3—C4—C5	179.58 (12)	N1—N2—C8—S1	172.89 (9)
C2—C3—C4—C5	−1.20 (19)	C8—N3—C9—C10	−97.70 (16)

### Hydrogen-bond geometry (Å, °)

**Table:**

*D*—H···*A*	*D*—H	H···*A*	*D*···*A*	*D*—H···*A*
O1—H1o···O2	0.83 (1)	2.26 (2)	2.717 (1)	115 (2)
O1—H1o···S1^i^	0.83 (1)	2.55 (1)	3.291 (1)	150 (2)
O2—H2o···O3	0.84 (1)	2.31 (2)	2.745 (1)	112 (2)
O2—H2o···O1^ii^	0.84 (1)	2.07 (1)	2.832 (1)	151 (2)
O3—H3o···S1^iii^	0.84 (1)	2.36 (1)	3.189 (1)	170 (2)
N2—H2N···S1^iv^	0.87	2.62	3.480 (1)	171

Symmetry codes: (i) −*x*+1, *y*−1/2, −*z*+1/2; (ii) *x*, −*y*+1/2, *z*−1/2; (iii) *x*, *y*, *z*−1; (iv) −*x*+1, −*y*+1, −*z*+1.
